# Biochemical diagnosis of mucopolysaccharidosis in a Mexican reference center

**DOI:** 10.1590/1678-4685-GMB-2018-0347

**Published:** 2020-02-14

**Authors:** Sandra del Carmen Mendoza-Ruvalcaba, Aniel Jessica Leticia Brambila-Tapia, Jesús Alejandro Juárez-Osuna, Thiago Donizete Da Silva-José, José Elías García-Ortiz

**Affiliations:** ^1^ Instituto Mexicano del Seguro Social, Centro de Investigación Biomédica de Occidente, Guadalajara, Jalisco, Mexico.; ^2^ Universidad de Guadalajara, Centro Universitario de Ciencias de la Salud, Departamento de Psicología Básica, Guadalajara, Jalisco, Mexico.; ^3^ Universidad de Guadalajara, Centro Universitario de Ciencias de la Salud, Guadalajara, Jalisco, Mexico.; ^4^ Instituto Mexicano del Seguro Social, Centro Médico Nacional de Occidente, UMAE Hospital Gineco-Obstetricia, Guadalajara, Jalisco, Mexico

**Keywords:** Mucopolysaccharidoses, leukocyte enzymatic activity, fluorometric assay, Mexico

## Abstract

Mucopolysaccharidoses (MPS) are a group of genetic disorders, each resulting from the deficiency of one of the lysosomal enzymes that catabolizes mucopolysaccharides. For the accurate diagnosis of the disease, the quantification of a specific enzymatic activity is needed. In the present study, we analyzed seven MPS over several periods of time ranging from 2 to 5 years in a reference center in Mexico. During this time, a total of 761 samples belonging to 505 individuals with suspected MPS were analyzed. A total of 198 (26.01%) positive results were found. Among these, MPS IVA accounted for the highest frequency of positive results (49.10%), followed by MPS III (17.69%, IIIA: 11.80% and IIIB: 5.89%). Adjusting for the number of births per year, the estimated incidence per 100,000 births for MPS analyzed were as follows: MPS I: 0.19, MPS II: 0.15, MPS IIIA: 0.26, MPS IIIB: 0.13, MPS IVA: 1.10, MPS VI: 0.17 and MPS VII: 0.23, and the combined estimated incidence of MPS was 2.23 per 100,000 births; however, this incidence seems to be highly underestimated when compared with the results of newborn screenings.

## Introduction

Mucopolysaccharidoses (MPS) are a heterogeneous group of rare inherited disorders, each caused by a deficiency of one of the lysosomal enzymes that catabolizes complex carbohydrates known as mucopolysaccharides or glycosaminoglycans (GAG). GAG accumulation can cause progressive damage to a broad range of tissues and can lead to clinical manifestations such as stiff joints, skeletal malformations, retarded growth, pulmonary deficits and ocular, hepatic, cardiac and neurological abnormalities (Muenzer, 2004). MPS are classified into seven different types (I, II, III, IV, VI, VII and IX), and MPS III and IV are further classified into four and two subtypes, respectively. In addition to the attenuated and severe phenotypes observed with almost all of the MPS types, the diagnosis of MPS is based on the enzymatic activities of leukocytes, fibroblasts or plasma (Cimaz and La Torre, 2014).

In this study, we present the initial results from a reference center in Mexico that evaluated the enzymatic activities of 7 different types of MPS (I, II, IIIA, IIIB, IVA, VI, and VII) over a period of 5 years (from 2012-2017).

## Subjects and Methods

Trained medical specialists from around the country sent samples from patients suspected of having an MPS and/or from their relatives. The enzymatic activities of the samples that were sent to the laboratory were examined over a period ranging from 2.7 to 4.9 years; the shortest period of 2.7 years was for MPS II, while the longest period of 4.9 years was for MPS I, the other MPS were examined between this range of time.

Leukocytes were extracted from the peripheral blood of the patients and parents (when requested) to obtain the leukocytes, which were subsequently homogenized by sonication. The proteins of the leukocytes were measured with the Lowry method (Lowry *et al.*, 1951) in a spectrophotometer (Beckman Coulter DU 730). The residual activities of each of the enzymes (MPS I, MPS II, MPS IIIA, MPS IIIB, MPS IVA, MPS VI and MPS VII) were measured using the fluorogenic substrate 4-methylumbelliferyl (4MU) as follows: the detection of the enzymatic activity in MPS I was performed according to the method of Ou *et al.* (2014), with the substrate 4MU-α-L-iduronide (Glycosynth®, code 44076); Moscerdam Substrates® were used for the analysis of MPS II, with the substrate 4MU-alpha-L-iduronide-2-sulphate (code M2) (Voznyi *et al.*, 2001); MPS IIIA, with the substrate 4MU-alpha-N-sulpho-D-glucosaminide (code M3A) (Karpova *et al.*, 1996); MPS IIIB, with the substrate 4MU-alpha-N-acetyl-glucosaminide (code M3B) (Marsh and Fensom, 1985); and MPS IVA, with the substrate 4MU–Beta-D-galactoside-6-sulphate (code M4A) (Van Diggelen *et al.*, 1990). All analyses were performed according to the manufacturers’ instructions. MPS VI was analyzed according to the methods of Kresse *et al.* (1982) with the substrate P-nitrocatechol sulfate dipotassium salt (Sigma®, code N-7251), and MPS VII was analyzed according to the method of Beaudet *et al.* (1975) with the substrate 4MU β-D glucuronide (Glycosynth®, code 44064). The tests were performed in a fluorometer (Turner 450, Sunnyvale, California) at an excitation wavelength of 360 nm and an emission wavelength of 415 nm. In the case of MPS VI, the residual activity analysis was performed with a Beckman Coulter DU 730 spectrophotometer (Brea, California). For each assay, a curve for 4MU or P-nitrocatechol (depending on the analysis) was constructed as a quality control; in addition, a control test with samples of healthy control subjects was performed; however, no positive controls were run in either assay.

Each protocol was standardized with at least 30 leukocyte samples of healthy individuals to determine the normal range for each enzymatic activity in the Mexican population (Table 1).

**Table 1:** Reference values in the healthy Mexican population.

MPS	Enzyme	Normal enzymatic activity values (range)
I	alpha-L-iduronidase	0.10-0.53 nmol/mg prot/h
II	alpha-L-iduronide-2-sulfatase	27.42-103.08 nmol/mg prot/min
IIIA	N-sulphoglucosamine-sulfohydrolase	0.89-3.47 nmol/mg prot/17 h
IIIB	N-acetyl-glucosaminidase	7.09-22.24 nmol/mg prot/17 h
IVA	N-acetylgalactosamine 6-sulfate –sulfatase	44.49-231.75 nmol/mg prot/18 h
VI	Arylsulfatase B	0.61-1.66 nmol/mg prot/min
VII	Beta-glucuronidase	63.40-200.47 nmol/mg prot/h

## Results

The ranges of the normal values are presented in Table 1. A total of 761 samples belonging to 505 different patients or relatives were examined. Among all samples, 409 (53.74%) were presented with requests for the detection of one enzymatic activity, 224 samples (29.43%) were presented with requests for tests of two different enzymes, 90 samples (11.82%) for three enzymes and 26 samples (3.41%) for more than three enzymes. For 12 samples (1.60%), requests included an MPS diagnosis plus a test for another disease. The samples were sent from virtually all of the 32 states in Mexico with the exception of Campeche, and Jalisco was the most frequent source of samples (27.40% of the analyses), followed by Chihuahua (12.10%) and Mexico City (6.80%); Tlaxcala, Zacatecas and Coahuila were the least frequent sources of samples (<1%).

When we analyzed the 761 samples according to sex, 45.6% and 52.8% of the samples were from females and males, respectively; however, in 1.6% of the samples, sex was not reported due to sample codification. The mean (range) age of the 443 (58.2%) individuals, for whom age information was available, was 7.73 (0-48) years. For 86.6% of the analyses, the samples were from the patients, 10% were from siblings and 3.4% were from parents. The most frequently requested MPS enzymatic assays were those for MPS IVA (31.41%) and MPS VI (23.26%), and the least frequently requested assay was for MPS II (5.65%).

A total of 198 (26.01%) positive results, from the 761 samples, were found. MPS IVA was associated with the highest frequency of positive results (49.10%), followed by MPS III (17.69%; IIIA: 11.80% and IIIB: 5.89%) and MPS VII (10.37%). The MPS with the lowest frequency of positive results was MPS II (6.76%). (Table 2).

**Table 2:** Data from probes performed for each type of MPS.

MPS	Availability of the test (years)	Samples N (%)	Positive results, N (%)	Percentage of positive results per MPS (positive results/samples)*100, (%)	Number of samples, adjusted for year (samples/years), N (%)	Number of positive results adjusted for year (positive results/years), N (%)	Incidence per 100 000 live births
I	4.9	120 (15.77)	23	19.17	24.49 (11.78)	4.69 (8.58)	0.19
II	2.7	43 (5.65)	10	23.25	15.93 (7.66)	3.70 (6.76)	0.15
IIIA	3.1	67 (8.80)	20	29.85	21.61 (10.40)	6.45 (11.80)	0.26
IIIB	3.1	69 (9.07)	10	14.49	22.26 (10.71)	3.22 (5.89)	0.13
IVA	3.8	239 (31.41)	102	42.68	62.89 (30.25)	26.84 (49.10)	1.10
VI	3.9	177 (23.26)	16	9.04	45.38 (21.83)	4.10 (7.50)	0.17
VII	3	46 (6.04)	17	36.96	15.33 (7.37)	5.67 (10.37)	0.23
Totals		761 (100)	198		207.90 (100)	54.67 (100)	2.23

With respect to the demographic data of patients, we observed that MPS VII had patients with the youngest age (4.33 years) and MPS IVA had the oldest age (11.23 years); no sex differences were observed, with the exception of MPS VII in which a high percentage of female patients with positive results was observed (80%) and MPS II, which according to its inheritance pattern, was observed only in males (Table 3). However, it is of interest that five of the 17 (29.41%) positive results for MPS VII were obtained from fetuses with the diagnosis of hydrops fetalis and five of the six (83.33%) patients with hydrops fetalis analyzed were positive for MPS VII.

**Table 3:** Demographic data for the analyzed patients.

MPS	Samples N (%)	Age in years, mean (range)	Male/female, N (%)
I	8	8 (3-16)	3 (37.5) / 5 (62.5)
II	10	8.87 (1-48)	10 (100) / 0 (0)
IIIA	9	11 (6-25)	4 (44.4) / 5 (55.6)
IIIB	7	6 (0-9)	4 (57.14) / 3 (42.86)
IVA	95	11.23 (0-42)	35 (36.84) / 60 (63.16)
VI	7	10.67 (2-17)	3 (42.86) / 4 (57.14)
VII	5	4.33 (0-12)	1 (20) / 4 (80)

Regarding the positive results according to the numbers of requested analyses per MPS type studied, we found the highest percentage of positive results were for MPS IVA (42.68%), and the combined MPS III subtypes (44.34%; IIIA: 29.85 and IIIB: 14.49%), followed by MPS VII (36.96%). MPS VI was associated with the lowest percentage of positive results (9.04%) (Table 2).

When we divided the positive results into patients (expected homozygous) and carriers (expected heterozygous) based on residual enzymatic activity (Table 4) and excluded MPS II (the only MPS with hemizygous patients), we observed that MPS IVA had the highest frequency of patients (93.14%), followed by MPS IIIB (70.00%). The lowest percentage of patients (expected homozygous) was observed in MPS VII (29.41%).

**Table 4:** Enzymatic activity values of patients (expected homozygous) and carriers (expected heterozygous).

MPS	Number of patients (%) a	Enzymatic activity values (mean ± SD) c	Number of carriers (%) b	Enzymatic activity values (mean ± SD) c	Total positive results	Normal enzymatic activity values
I	8 (34.78)	0.009 ± 0.008	15 (65.22)	0.047 ± 0.02	23 (100)	0.10-0.53
II	10 (100)	4.03 ± 4.34	0 (0)	-	10 (100)	27.42-103.08
IIIA	9 (45)	0.04 ± 0.11	11 (55)	0.49 ± 0.19	20 (100)	0.89-3.47
IIIB	7 (70)	-0.07 ± 0.58	3 (30)	2.56 ± 1.06	10 (100)	7.09-22.24
IVA	95 (93.14)	-6.62 ± 11.34	7 (7.86)	22.54 ± 2.58	102 (100)	44.49-231.75
VI	7 (43.75)	0.02 ± 0.06	9 (56.25)	0.24 ± 0.11	16 (100)	0.61-1.66
VII	5 (29.41)	1.28 ± 2.09	12 (70.59)	27.67 ± 13.21	17 (100)	63.40-200.47
**Totals**	141 (71)		57 (29)		198 (100)	

a The following values were taken for patients (expected homozygous) based on clinical and molecular data when available: MPS I ≤ 30% of residual enzymatic activity (REA), MPS II ≤ 40% of REA, MPS IIIA ≤ 23% of REA, MPS IIIB ≤ 10% of REA, MPS IVA ≤ 27% of REA, MPS VI ≤ 10% of REA, and MPS VII ≤ 10% of REA.

b The following values for the residual enzymatic activity were taken for carriers (expected heterozygous): MPS I: 31-77% of REA, MPS IIIA: 24-85% of REA, MPS IIIB: 20-50% of REA, MPS IVA: 40-60% of REA, MPS VI: 11-61% of REA and MPS VII: 18-83% of RAE.

c MPS I and MPS VII were measured as nmol/mg prot/hr, MPS II and MPS VI as nmol/mg prot/min, MPS IIIA and IIIB as nmol/mg prot/17 hr, and MPS IVA as nmol/mg prot/18 hr.

The estimated incidence of each MPS was calculated by dividing the positive results adjusted by year by the birth rate in Mexico and multiplied by 100,000 (average from 2012 to 2015: 2,448,695). The combined estimated incidence of the 7 types of MPS detected in this study was 2.23 per 100,000 live births. This estimated incidence was obtained based on the sum of all of the estimated incidences (Table 2). However, this estimated incidence might have been underestimated considering that the diagnosis was performed by a geneticist referral and not by a newborn screening, which is not routinely performed in Mexico.

## Discussion

To the best of our knowledge, this is the second report that presents enzymatic activity results related to most MPS types based on samples from subjects recruited from a reference center in Mexico. Relative to the report of Zetina and González-Noriega (1989), in the present study, we analyzed approximately 5 times more patients in the same period of time and detected approximately 5 times more positive results (198 vs 35). In the previous report, the most frequently detected MPS (MPS IVA) coincided with our results. This high frequency suggests that MPS IVA is the most frequently diagnosed MPS in Mexico; however, the rest of the MPS types were not coincident with this report, and these differences are attributed to the larger sample size in the present study, which better represents the Mexican population. These results differ from results that have been reported in other countries, in which MPS IVA is, at most, the second-most frequently diagnosed MPS (Khan *et al.*, 2017). However, the determination of GAGs was not performed in patients, so the studied patients may have another MPS type not determined in the study; therefore, we generalized the results only to the analyzed types.

Additionally, we found that MPS IVA, MPS IIIA, MPS VII and MPS I were the most frequently diagnosed MPS types; this pattern of frequency is not similar to any reported pattern in other countries (Khan *et al.*, 2017). However, when comparing the combined estimated incidences of MPS types observed in this study with those observed in other countries, we found that the results were similar to those reported in Tunisia (2.27), which is located more or less in the middle of the list (ordered by frequency) of countries reported by Khan *et al.* (2017); however, this incidence should be higher considering that not all MPS types were evaluated in this study and that the results were not obtained from a nationwide newborn screening, which is more accurate. In addition, a recent study in Mexico reporting newborn screening (performed with multiplex tandem mass spectrometry in dried blood spots) for six lysosomal storage disorders that included MPS I (Navarrete-Matínez *et al.*, 2017) showed a much higher incidence per 100,000 births for this MPS (9.99) than the reported here (0.19); likewise, a pilot study of newborn screening in the United States reported an even higher incidence per 100,000 for MPS I (13.6) (Elliott *et al.*, 2016). These results highlight the underestimation of these conditions by routinely performed methods, which consists of the analysis of suspected individuals.

Thus far, enzyme replacement therapy for five MPS types (I, II, IVA, VI and VII) is available, and treatments for other MPS types are under development (Cimaz and La Torre, 2014); therefore, early diagnosis by a neonatal screening, such as via multiplex tandem mass spectrometry (Lawrence *et al.*, 2014; Donati *et al.*, 2018), is necessary and would provide a better incidence of these conditions.

Regarding the best diagnosed MPS, we found that MPS IVA was associated with the highest frequency of positive results from all of the analyses performed for MPS IVA (42.68%). This result could indicate that this MPS is easier to diagnose for Mexican clinicians, which is probably due to its characteristic phenotype or because this type is better known than other types.

Regarding age, we observed that most of the MPS types had similar average ages. Regarding sex, with the exceptions of MPS VII and MPS II, MPS analyses were positive with similar frequencies among males and females. MPS II was observed only in males, which is explained by its X-linked recessive inheritance pattern. The high percentage of females with positive MPS VII analyses (including carriers and patients, 80%) could be a random finding considering that previous reports have observed a higher prevalence of male patients (Montaño *et al.*, 2016; Zielonka, *et al.*, 2017). The high percentage of hydrops fetalis observed in positive patients and carriers for MPS VII (29.41%) coincides with previous reports that show that 41.1%-45.5% of MPS VII patients have a history of hydrops fetalis (Montaño *et al.*, 2016; Zielonka *et al.*, 2017). It is possible that the percentage observed in this study was underestimated due to the lack of information on the history of hydrops fetalis in other positive patients. Likewise, the high percentage of positive results for MPS VII among the patients analyzed with hydrops fetalis (83.33%) highlights the importance of performing this test in patients with hydrops fetalis.

In conclusion, we report the initial results from a national reference center regarding MPS in Mexico from which we detected the estimated incidences of most MPS types as well as the overall estimated incidence of MPS from all over the country. The high estimated incidence of positive results for MPS IVA is of interest; this type was also the most accurately diagnosed and presented the highest frequency of patients versus carriers; these findings differ from findings reported from other countries in which MPS IVA is, at most, the second-most frequently diagnosed MPS. Regarding the combined estimated incidence, we observed that incidence in Mexico is in the middle of the reported countries, and this frequency may be underestimated.

## Conflict of interest

The authors declare that there are no conflicts of interest that could be perceived as prejudicial to the impartiality of the reported research.

## Author contributions

JEGO conceived the study, SCMR conducted the experiments, AJO conducted the experiments, TDDSJ conducted the experiments and AJLBT analyzed the data and wrote the manuscript. All authors read and approved the final version of the manuscript.
